# Differentiation and Migration of Bone Marrow Mesenchymal Stem Cells Transplanted through the Spleen in Rats with Portal Hypertension

**DOI:** 10.1371/journal.pone.0083523

**Published:** 2013-12-10

**Authors:** Song Sun, Gong Chen, Menghua Xu, Yingli Qiao, Shan Zheng

**Affiliations:** Surgical Department, Children’s Hospital of Fudan University, Shanghai, China; Wake Forest Institute for Regenerative Medicine, United States of America

## Abstract

**Aims:**

The goals of this paper were to evaluate the differentiation of bone marrow mesenchymal stem cells (BMSCs) into hepatocyte-like cells *in* *vitro*, and to determine whether stem cells can migrate and plant into the liver with portal hypertension accompanied by the end-stage of liver disease.

**Methods:**

BMSCs were isolated from rats and amplified with hepatocyte growth factor (HGF) and fibroblast growth factor-4 (FGF-4). The expression of alpha-fetoprotein (AFP), cytokeratin 18 (CK-18), and albumin (ALB) was detected by immunofluorescence in induced cells. Rats were randomly divided into experimental (with common bile duct ligation) and control groups. After injection of fluorescence labeled cells, cell distribution was observed under a fluorescence microscope. The integrated optical density (IOD) and cell distribution scores were evaluated using Image-Pro Plus 6.0 software. The portal pressure was measured before the rats were killed.

**Results:**

After being induced with HGF and FGF-4, the Golgi apparatus, endoplasmic reticulum, ribosomes, and mitochondria all significantly increased in the fifth generation cells. Immunofluorescent analysis showed that the induced cells expressed AFP, CK-18, and ALB. BMSCs were stained by CM-Dil, and the labeling rate was as high as 95.5%. The portal pressure in experimental group was much higher than that of the control group (18.04±2.35 vs. 9.75±1.40cm H_2_O *p*<0.01). The IOD of transplanted cells in the experimental group was also significantly higher than that of the control group (11.30±2.09×10^5^ vs. 2.93±0.88×10^5^, *p*<0.01). In addition, the cell distribution score in the experimental group was lower than that of the control group (1.99±0.36 vs. 2.36±0.27, P<0.05).

**Conclusions:**

The combination of HGF and FGF-4 induces the differentiation of BMSCs into hepatocyte-like cells, which express AFP, CK-18, and ALB. In addition, the recruitment of BMSCs (after transplantation in the spleen) was improved in rats with portal hypertension.

## Introduction

Cholestasis hepatic cirrhosis is a serious clinical problem that cannot be reversed. Liver transplantation is the only way to improve the long-term prognosis of end-stage liver cirrhosis [1-3]. However, the clinical application of organ transplantation has challenges due to donor shortage, high operation expenditure, and post-transplantation complications. Considering these challenges, hepatocyte transplantation has been suggested as a good choice because of the simpler and less invasive procedure; potentially, one donor can serve multiple recipients. However, studies have shown that less than 20%–30% of transplanted hepatocytes survive upon transplantation [4]. Furthermore, the source of hepatocytes is scarce, and the limited replicative capacity of these cells is also a problem. Thus, new approaches are needed. 

Mesenchymal stem cells (MSCs) are a stem cell population within the bone marrow, which have been shown to have increasing therapeutic potential in a wide range of diseases [5-8]. Bone marrow mesenchymal stem cell (BMSC) transplantation has been an important area of research for the treatment of terminal stage liver diseases since it was shown for the first time in 1999 that BMSCs can differentiate into hepatocytes and bile duct epithelial cells [9], a finding that since been supported by other studies [10-12]. Furthermore, other scientists have advocated that BMSCs might be the most appropriate source for the generation of hepatocytes [13,14]. A number of factors, including HGF and FGF-4, have been considered important for the formation of hepatocytes [15-17]. In some centers, BMSCs have also been used in clinical trials [18,19]. However, since liver diseases are often accompanied by portal hypertension, there exists the challenge of whether the transplanted cells can migrate and plant into the liver in the condition of portal hypertension

The goal of this study was to elucidate the hepatic differentiation potential of rat BMSCs, explore the feasibility of treating end-stage liver disease by transplanting BMSCs through the spleen, and stimulate the migration and recruitment of BMSCs in the presence of portal vein hypertension.

## Materials and Methods

### Reagents

We purchased the following reagents as follows: LG-DMEM culture medium (Sigma Aldrich, St. Louis, MO), fetal bovine serum (Gibco Life Technologies, Grand Island, NY), rabbit polyclonal to alpha-1 fetoprotein (Abcam, Cambridge, MA), mouse monoclonal to cytokeratin-18 (Abcam), sheep polyclonal to albumin (Abcam), fluorescein (FITC) goat anti-rabbit IgG (Jackson ImmunoResearch Laboratories, Inc, West Grove, PA), rhodamine (TRITC) goat anti-mouse IgG (Jackson), fluorescein (FITC) donkey anti-sheep IgG (LifeSpan BioSciences, Seattle, WA), recombinant human hepatic growth factor (rhHGF, PeproTech, Oak Park, CA), recombinant human fibrous growth factor-4 (rhFGF-4, PeproTech), and chloromethyl-benzamide dialkyl carbocyanine fluorescent dye (CM-Dil, Molecular Probes, Eugene, OR).

### Isolation, culture, and differential induction of BMSCs

Bone marrow was harvested by flushing the tibiae and femurs of 200 g Sprague-Dawley rats with 1x phosphate buffer saline (1x PBS), after which they were filtered through a strainer, and suspended in Dulbecco's Modified Eagle's Medium (DMEM, Sigma) supplemented with 15% fetal bovine serum (GIBCO/BRL) [20]. Cells were incubated at 37°C in 5% humidified CO2 for 24 h as primary culture until the medium was replaced to remove non-adherent cells. When cells reached 90% confluence, they were trypsinized with 0.25% trypsin for 1 min at 37°C. After centrifugation, cells were re-suspended with serum-supplemented medium and incubated in 25 cm^2^ culture flasks. The resulting cultures were referred to as first-passage cultures [21,22]. When the cells were expanded to fifth-passage, both rhHGF (50 ng/ml) and rhFGF-4 (10 ng/ml) were added to the medium [21,23-25]. The cells were cultured for 2 more weeks, and then harvested for detection and transplantation.

### Scanning electron microscopy

First-passage, fifth-passage, and induced fifth-passage cells were collected, and cell aggregates were obtained through centrifuging. The specimens were pre-fixed in a 2.5% solution of isotonic buffered glutaraldehyde, followed by post-fixation in a 1% osmium tetroxide solution. After uranylacetate staining, the specimens were embedded in Epon and cut in thin sections (50 nm) using an ultramicrotron. The samples were observed by transmission electron microscopy at a voltage of 80 kV (Philips CM120, Holland). Cytoplasmic organelles were compared among the three kinds of cells.

### Immunofluorescence (IF) analysis

After 2 weeks of induction, cells were detected as described [26]. Briefly, cells that adhered to the glass slides were washed twice with cold PBS (1x), and fixed in 4% paraformaldehyde for 20 min at 4°C. Following three washes with PBS (5 min each), cells were permeabilized with 0.2% Triton X-100 for 30 min, and blocked in 2% BSA for 30 min. Then, the cells were incubated overnight at 4°C with rabbit polyclonal to alpha-1 fetoprotein (1:200), mouse monoclonal to cytokeratin-18 (1:200), or sheep polyclonal to albumin (1:200). The cells were then washed with PBS three times (5 min each) and incubated for 50 min at room temperature with FITC-conjugated goat anti-rabbit IgG secondary antibody (1:2000), TRITC-conjugated goat anti-mouse IgG secondary antibody (1:2000) or FITC-conjugated donkey anti-sheep IgG (1:1000). Subsequently, the cells were stained with diamidinophenylindole (DAPI) for 5 min at room temperature and observed under a fluorescence microscope (Leica, Germany).

### Cell labeling

Cells were labeled as previously described [27]. Stock solutions of CM-Dil were prepared in dimethylsulfoxide (DMSO) at 1 g/L. Immediately before labeling, the 1g/L stock solution was diluted with Dulbecco’s PBS (D-PBS) to a working concentration of 2 mg/L. The cells were resuspended in the working solution and incubated for 5 min at 37°C, and then for an additional 15 min at 4°C, after which they were rinsed twice with PBS. The labeled cells were resuspended in PBS at a density of 1x10^6^ cells/ml, and prepared for transplantation. The labeling efficiency was counted.

### Animal Models

We used Sprague-Dawley rats (Department of Laboratory Animal Science, Fudan University, China). Animals were kept in a temperature-controlled environment (22°C) with 12:12-hour light-dark cycles and free access to water and standard chow. All experimental protocols were approved by and performed according to the Animal Committee of Fudan University. The Sprague-Dawley rats (200 g) were randomly divided into 2 groups. Before surgery, animals were anesthetized with chloral hydrate (250 mg/kg BW) intraperitoneally. Briefly, a short midline abdominal incision was made and the common bile duct was exposed and isolated. The common bile duct of the rats (n=10) was subjected to double ligation with a 4-0 silk suture, and resected between the two ligatures. Sham-operated rats (n=8) served as controls. In these rats, the common bile duct was exposed by only a median laparotomy, but neither ligation nor resection was performed. Two weeks after the surgery [28], the animal model of obstructive liver disease with portal hypertension was developed.

### Cell transplantation

The abdomen of the rats was reopened, and the induced and labeled cells (resuspended in 1x PBS at a density of 1×10^6^ cells/ml) were injected via the lower pole of spleen into the recipients in the volume of 1 ml. After cell injection, the lower pole of spleen was immediately ligated to prevent bleeding and reflux of cells. The cells (marked by CM-Dil) were kept in the dark during transplantation. The recipient rats were kept for 1 week.

### Measurement of portal vein pressure

After BMSC transplantation, all rats were kept for more than 1 week. Then, the rats were anesthetized with chloral hydrate (250 mg/kg) intraperitoneally, and an abdominal incision was made at the right upper quadrant. Without taking out the liver, an 18-gauge cannula was inserted into the portal vein, which was connected to a SLY-B electrophysiolograph via a strain-gauge transducer. The portal pressure was then monitored continuously. It took approximately 5 to 10 min for the pressure to become stable, after which the pressure was recorded for another 1 min. The portal pressure of the two groups was analyzed by a t-test. 

### Fluorescence measurement of frozen liver sections

After measurement of portal pressure, the thoracic cavity was opened and the heart was exposed. Then, the left ventricle was punctured and indwelled with an 18-gauge cannula. The right atrial was then opened and a large volume of saline was infused quickly until the liver turned pallor. Subsequently, 4% paraformaldehyde was slowly infused. A portion of the right liver lobe (50 mg) excised from each liver was fixed in 4% paraformaldehyde, and then embedded in paraffin. Frozen sections were prepared by a routine method in the dark (1 tissue: 10 sections). Liver sections were randomly assessed and photographed and coded under a fluorescence microscope. The fluorescent microscope images were assessed using Image-Pro Plus 6.0 software, and the integral optical density (IOD) of each photograph was collected. The IOD between the two groups was compared by the t-test.

## Results

### Isolation and culture of mesenchymal stem cells from rat bone marrow

**Figure 1:**
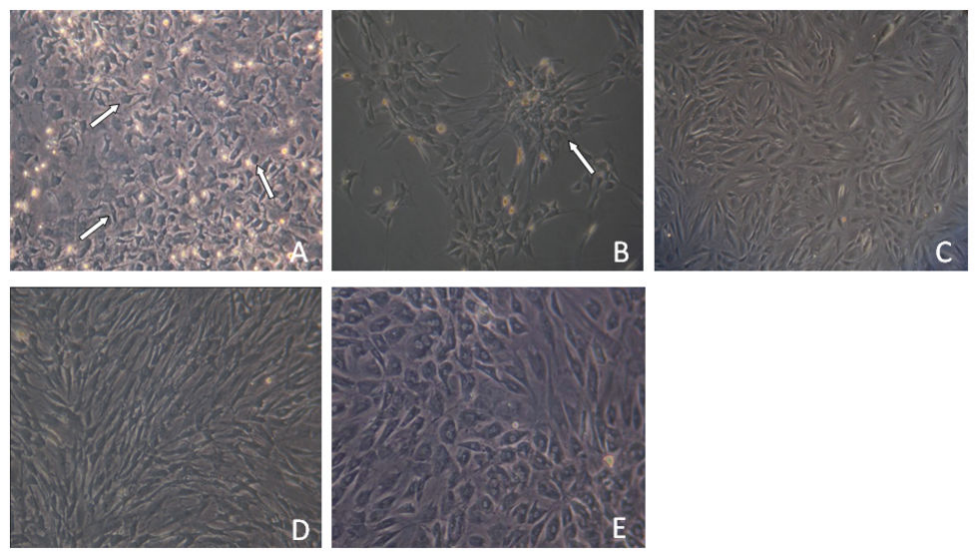
Cell morphology and arrangements in different generations. (A) Primary cells show morphological diversity, and present irregular polygon, round, spindle; (B) After passage, the cells show colony-like growth and their shape gradually becomes consistent as the cell purity increases; (C) After 5 passages, the spindle cells were highly homogeneous with few cell impurities; (D) Cells of 90% confluence arranged into a spiral shape; (E) After induction with HGF and FGF-4 for 2 weeks, the cells gradually become larger and cuboidal in shape. 100x.

**Figure 2:**
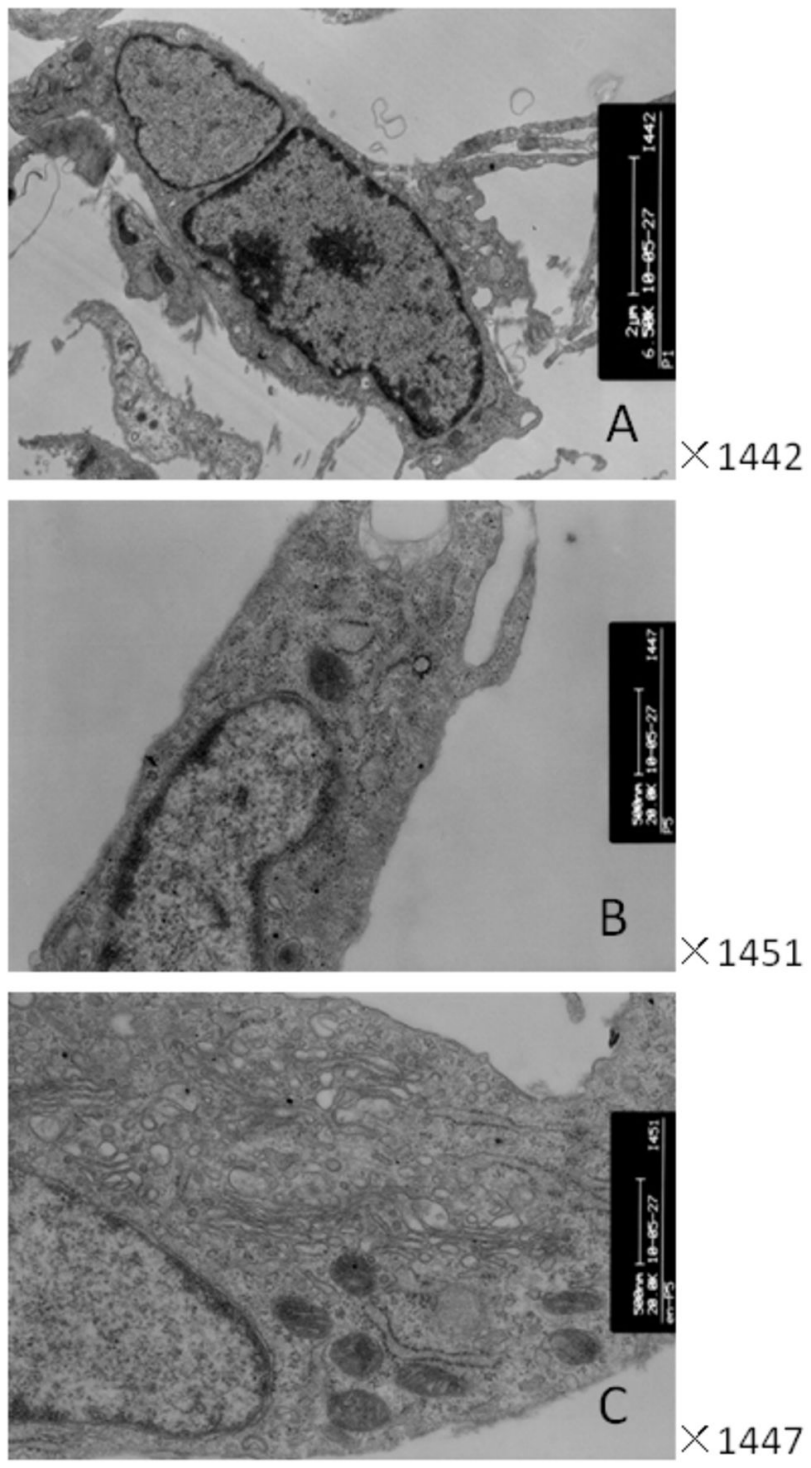
Transmission electron micrograph of BMSCs. Cells of 1^st^ (A) and 5^th^ (B) generation were much smaller and with less organelles; however, the cells induced at 5^th^ (C) generation were much larger and displayed organelles like Golgi bodies, ribosomes, and endoplasmic reticulum.

Plastic-adherent BMSCs were successfully isolated and expanded. BMSCs in passage 1 presented with a polygonal shape or anomalous formation with adherence to plastic and organization in a monolayer. After BMSCs were subcultured to the next passage, many colony units were formed, and gradually all fused to the plane. In the fifth passage, BMSCs spread out in appearance and acquired a spindle-shaped morphology (Figure 1). No significant differences in organelles between the first and fifth passage cells were noted under a transmission electron microscope. However, the Golgi apparatus, ribosomes, and endoplasmic reticulum increased significantly in the fifth passage cells induced by both rhHGF and rhFGF-4 (Figure 2).

### IF Analysis

**Figure 3:**
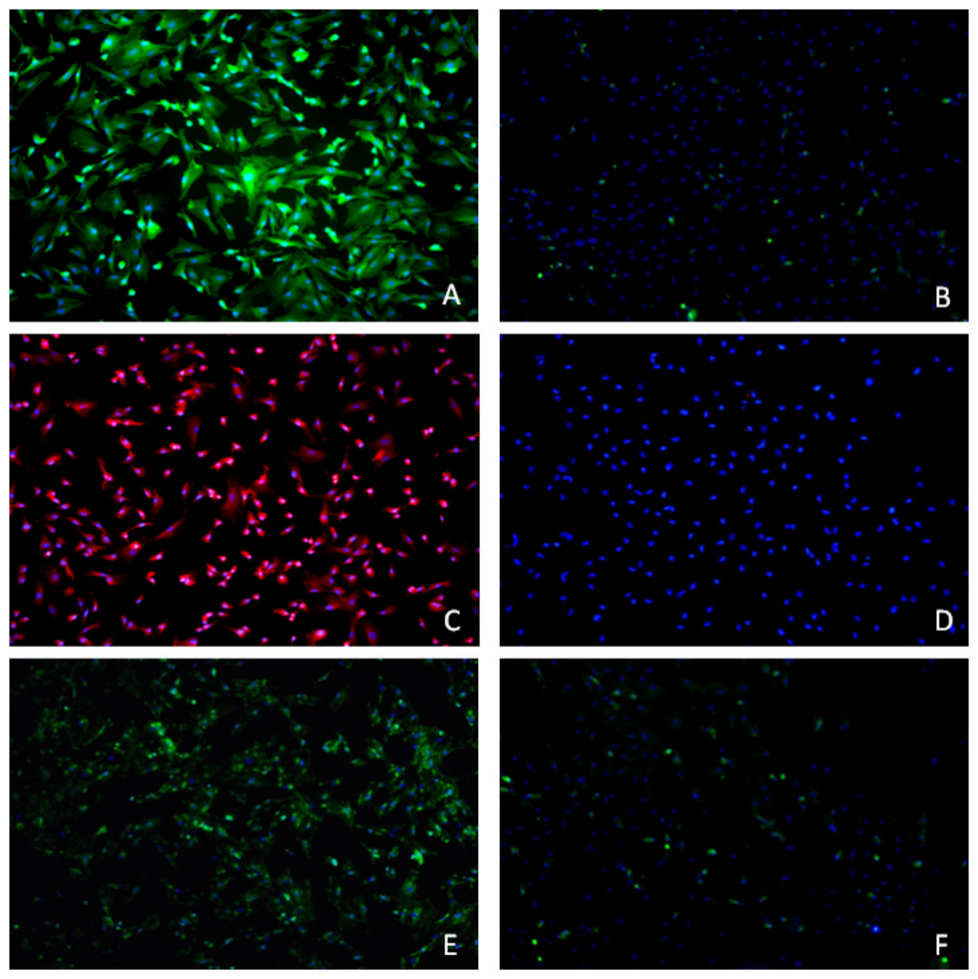
Immunofluorescence of HGF and FGF-4 induced BMSCs. The green fluorescence shows AFP expression, which is much higher in induced cells (A) than in non-induced cells (B). The red fluorescence shows CK-18 expression, which is much higher in induced cells (C) than in non-induced cells (D). The green fluorescence shows ALB expression, which is higher in induced cells (E) than non-induced cells (F). 100×.

IF analysis showed that the cells without induction were negative for AFP, CK-18, and ALB, whereas cells incubated for another 2 weeks in medium with rhHGF and rhFGF-4 stained positively for all of these markers (Figure 3).

### Cell Labeling

**Figure 4:**
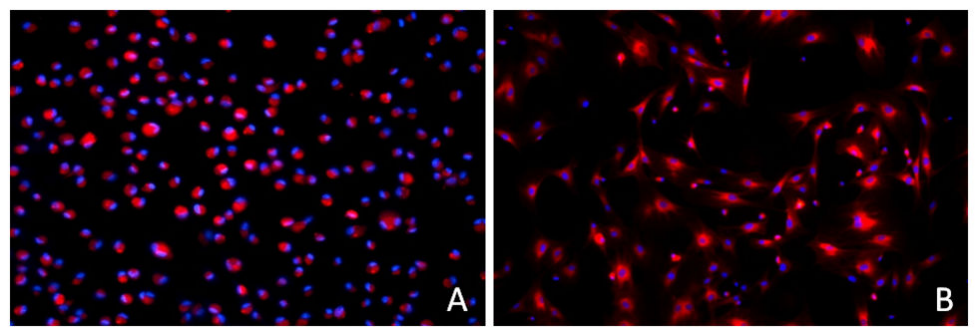
Fluorescence micrograph of CM-Dil labeled cells. More than 95% of cells exhibited red fluorescence (A). When the labeled cells were cultured *in* *vitro* again, most of the cells were able to grow while adhering to the wall (B), which indicated that the labeled cells were able to survive well.

The CM-Dil-labeled cell membrane showed a red fluorescence under a fluorescence microscope, and all nuclei were stained with DAPI. Cells and nuclei were counted in 10 microscopic fields. The labeling rate of CM-Dil was 95.5% (Figure 4A), and most of the labeled cells displayed a normal morphology (Figure 4B).

### Portal Pressure and Cell Planting

**Figure 5:**
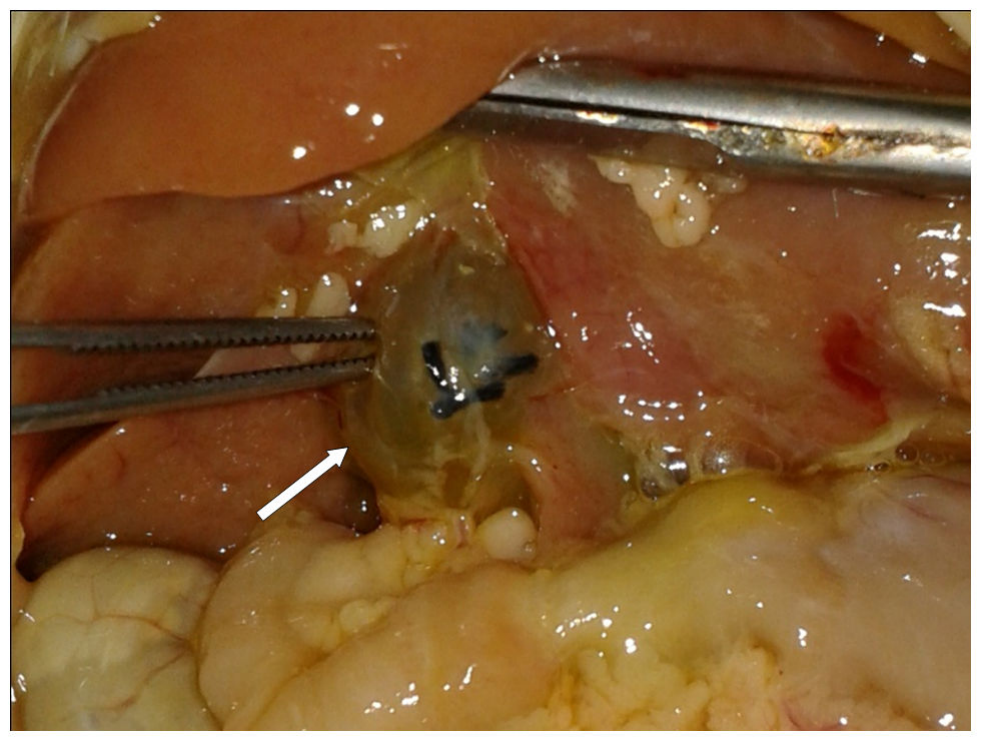
The cyst at porta hepatis. The common bile duct was ligated and the rats were maintained for 2 more weeks. Cysts were developed at the porta hepatis of all rats in the experimental groups. Internal organs such as the liver, intestine, and omentum were stained yellow as indices of cholestasis.

**Figure 6:**
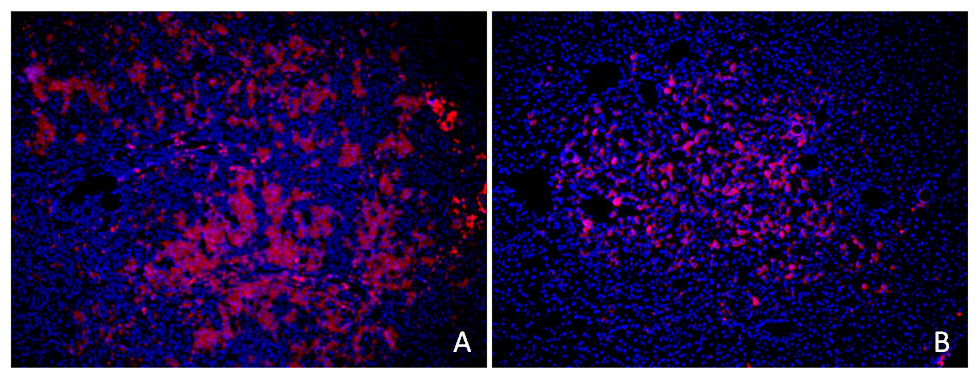
The distribution of fluorescence labeled BMSCs. The labeled cells migrated to the liver in the experimental group (A) were higher in number compared to those in the control group (B). The labeled cells were unevenly distributed and separated in different regions of the experimental groups (A). In the control group, the transplanted cells were distributed evenly throughout the field. 100x.

Of the 18 rats, only one died 3 hours after cell transplantation. Two weeks after common bile duct ligation, there were a series of clinical manifestations of biliary obstruction, including auricle and tail jaundice, deep yellow urine, off-white stool, and emaciation. All surviving rats in the experimental group had cysts at the porta hepatis (Figure 5); and, all livers were clearly enlarged and had sclerosis with many nodules. The portal pressure of the experimental group was significantly higher than that of the control group (18.04±2.35 vs. 9.75±1.40, cm H_2_O *p*<0.01) (Table 1). The IOD of the experimental group was much larger than that of the control group (11.30±2.09×10^5^ vs. 2.93±0.88×10^5^, *p*<0.01), which showed that more cells migrated and planted into the liver of the experimental group (Table 1). However, the cells were more evenly distributed in the microscopic field of the control group, whereas they were separated in different areas by fibrous tissue in the experimental group (Figure 6). 

**Table 1:** Portal vein pressure (PVP) and IOD of transplanted cells.

Experimental group	PVP (cmH_2_O)	IOD	Control group	PVP (cmH_2_O)	IOD
1	17.7	1180444	1	9.7	341905.5
2	16.9	1265803	2	11.5	231085.1
3	15.3	1029792	3	10.1	214956
4	16.4	709806.7	4	11.3	299649.2
5	23.4	1241142	5	7.8	391162.4
6	19.4	1068480	6	8.4	391162.4
7	18.1	1230643	7	10.7	151112
8	16.7	998500.2	8	8.5	402792
9	18.5	1445993			
mean	18.04±2.35	1130067±209464		9.75±1.40	293505±88383

## Discussion

Studies have shown that BMSCs can be easily isolated and proliferate, can be autotransplanted without immunogenic reaction, are prone to differentiate into hepatocyte-like cells, and migrate into the liver [14,29]. A recent study suggested that BMSCs have a potential therapeutic effect against fibrosis [30-35] via inhibition of collagen formation. *In vivo* and *in vitro* experiments have demonstrated that BMSCs stimulate hepatocyte regeneration [36,37]. The advantage of using BMSCs may be a key therapeutic approach in cell replacement therapy in end-stage liver diseases. Most studies have focused on the induction of BMSC differentiation into hepatocyte-like cells, and the curative effect of transplantation; however, the portal hypertension associated with hemodynamic changes in the cell migration of BMSCs transplanted through the spleen has seldom been investigated. In this study, the isolation, culture, differential induction, and fluorescence labeling of BMSCs were performed. In addition, we studied the portal hypertension hemodynamics in cell migration and planting of BMSCs transplanted through the spleen. BMSCs can be purified through cell passage because of its anchorage-dependent growth character. Studies have confirmed that the purity reaches up to 90% when the cells are in their third [22], which meets the transplantation needs. In our study, we used fifth generation cells.

There are no standard methods for inducing BMSCs. However, HGF combined with FGF, EGF, and/or oncostain M are frequently used for 2 or 3 weeks to induce the differentiation of BMSCs into hepatocyte-like cells [21,23–25]. In this study, we cultured BMSCs in solution with HGF and FGF-4 for 2 weeks before detecting the expression of AFP and CK-18, which are expression products of immature hepatocytes [38]. AFP is also expressed in germ cell tumors [39], whereas CK-18 is also expressed by accessory glands of the skin, and the epithelial neoplasm of some digestive organs and urocysts [40]. None of these protein markers are expressed in primary cell culture, which indicates that part of the liver’s excretory function is gradually generated during the course of passage and induction [26]. Furthermore, in respect to cell structure, organelles such as Golgi bodies, reticulum, ribosomes, and mitochondria increased significantly after induction. This change might be an indication that the cells have transitioned to a more active synthetic and secretory state, potentially indicative of the differentiation of BMSC into hepatocyte-like cells. However, further studies are needed to determine if there is indeed a correlation between morphological and functional changes.

There are many ways to transplant BMSCs, including an IV push via through the portal and caudal veins, as well as injection into the spleen, liver, and peritoneum. Other studies have shown that the hepatocytes transplanted through the spleen exhibit good physiological function and high long-term viability, and can migrate to the liver [41]. These advantages might profit from the following characteristics that the spleen develops. For example, the big space in the splenic sinusoid is able to store transplanted cells. The reticular tissue in the splenic red pulp allows cellular interactions, which can induce immune tolerance. In addition, there might be less cell mass to embolize the portal vein system after splenic sinusoid filtration. Thus, we used this approach to transplant stem cells into the rats. CM-Dil, a lipophilic fluorescent dye, is easily embedded in the cell membrane and diffuses laterally, thereby marking the entire cell membrane. CM-Dil can also go into the daughter cell membrane along with segmentation [42]. Therefore, CM-Dil is an excellent cell dye to be used. In addition, we used IOD instead of fluorescent cell counting to avoid having cell division affect our results. 

Some studies have suggested that hepatic fibrosis and portal hypertension may block the migration of BMSCs to the liver after cell transplantation [43]. On the other hand, other studies support the concept that the injured liver may release some chemicals to recruit BMSCs [31,44,45]. Clinical trials have also shown [46] that the levels of HGF and TGF-α increased in the serum of patients with acute liver injury. HGF, SDF-1, and MMP-9 were also upregulated in the injured liver, a finding that suggests that the injured liver may synthesize some chemokines that stimulate the cells to migrate and plant into the liver [44]. Our study further confirms that cells transplanted through the spleen were susceptible to migration and transplantation into the injured liver. We speculate that there may be some powerful induce factors in the liver, which can make stem cells migrated into liver against the higher blood pressure. With regard to cell distribution, cells were unevenly distributed in different regions of the injured liver. However, in the control group, the transplanted cells were distributed evenly throughout the liver. This may be due to the movement of transplanted cells that was blocked by fibroblastic proliferation during the course of cirrhosis.

In conclusion, the BMSCs that migrated into the injured liver were greater in number than those observed in the normal liver, even though portal pressure was higher than normal rats. However, the distribution of cells was more uneven in the injured liver. These results provide a theoretical basis for the clinical application of hepatic stem cell transplantation. However, whether BMSCs can survive long-term *in vivo* and produce therapeutic effects, needs to be further evaluated.
